# Bioglasses Versus Bioactive Calcium Phosphate Derivatives as Advanced Ceramics in Tissue Engineering: Comparative and Comprehensive Study, Current Trends, and Innovative Solutions

**DOI:** 10.3390/jfb16050161

**Published:** 2025-05-03

**Authors:** Monika Furko

**Affiliations:** Institute of Technical Physics and Materials Science, Centre for Energy Research, Konkoly-Thege Str. 29-33, H-1121 Budapest, Hungary; furko.monika@ek.hun-ren.hu

**Keywords:** bioceramics, bioglasses, tissue engineering, calcium phosphates, biocompatibility, biodegradability, scaffolds, injectable hydrogels

## Abstract

Tissue engineering represents a revolutionary approach to regenerating damaged bones and tissues. The most promising materials for this purpose are calcium phosphate-based bioactive ceramics (CaPs) and bioglasses, due to their excellent biocompatibility, osteoconductivity, and bioactivity. This review aims to provide a comprehensive and comparative analysis of different bioactive calcium phosphate derivatives and bioglasses, highlighting their roles and potential in both bone and soft tissue engineering as well as in drug delivery systems. We explore their applications as composites with natural and synthetic biopolymers, which can enhance their mechanical and bioactive properties. This review critically examines the advantages and limitations of each material, their preparation methods, biological efficacy, biodegradability, and practical applications. By summarizing recent research from scientific literature, this paper offers a detailed analysis of the current state of the art. The novelty of this work lies in its systematic comparison of bioactive ceramics and bioglasses, providing insights into their suitability for specific tissue engineering applications. The expected primary outcomes include a deeper understanding of how each material interacts with biological systems, their suitability for specific applications, and the implications for future research directions.

## 1. Introduction

Bioactive materials (bioglasses and ceramics) are commonly employed to restore and replace unhealthy and damaged hard and soft tissues [1]. Two major classes of bioactive materials are bioactive ceramics and bioglasses. They have gained widespread attention due to their ability to connect with biological tissues, promote cellular interactions, and regenerate tissue [2]. Thus, these novel materials are pivotal in a wide range of biomedical applications. According to the host tissue interactions, bioceramics can be classified as nearly bioinert, bioactive, and bioresorbable [3]. Among these, calcium phosphate-based ceramics are highly bioactive materials, and they are particularly significant due to their compositional similarity to natural bone minerals. Overall, the damaged tissues and bones can be restored or replaced using these types of biomaterials and other bioactive molecules. Theoretically, bioceramics and bioglasses bond to bone through a bone-like carbonated hydroxyapatite layer, facilitating effective biological interaction [4]. Similarly, bioglasses are also recognized for their high bioactivity and potential stimulation of osteogenesis [1]. The advantages of bioactive ceramics include their various chemical compositions and controlled degradation rates, whereas bioglasses excel in forming strong bonds with hard tissues and have enhanced versatility in composition. However, both materials present challenges such as limited flexibility, fragile texture, and low mechanical properties [5,6,7]. It is well known that the most bone-like bioactive ceramics are the different calcium phosphate phases, such as hydroxyapatite (HAp), dicalcium phosphate (DCP), and tricalcium phosphate (TCP), that are widely used in bone tissue engineering since they can support osteointegration and osteoconduction. On the other hand, bioglasses, such as 45S5 Bioglass, are known for their ability to bond with soft and hard tissues, making them versatile for widespread applications such as bone grafts, scaffolds, coatings for implants, and even in dentistry as bone fillers. However, their brittleness (in ceramics) and rapid degradation (in bioglasses) limit their standalone use or their applications in load-bearing conditions. Recent advancements have focused on combining these materials with biopolymers to overcome these limitations [8,9].

**Figure 1:**
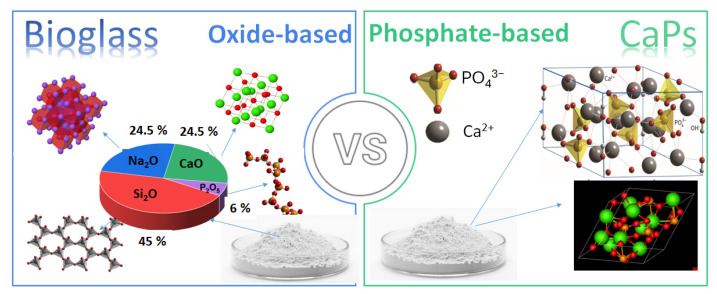
Schematic illustration the differences in compositions of bioglasses and CaP-based ceramics.

In addition, the demand for biomaterials in regenerative medicine has also significantly increased due to the growing need for functional tissue replacements. Consequently, recent research has expanded their use in soft tissue repair [10], wound healing [11,12], and drug delivery [13,14,15]. Despite extensive research on both material types, a detailed comparative analysis of calcium phosphate-based ceramics vs. bioglasses in tissue engineering is still missing. This review systematically compares their biological interactions, material properties, applications, and limitations to provide a clear understanding of their respective advantages and potential improvements. The review also explores composites with biopolymers, which offer enhanced mechanical and biological properties, making them attractive alternatives for clinical applications. Figure 1 illustrates the differences between the two materials in composition and main constituents.

## 2. Calcium Phosphate-Based Bioactive Ceramics

Calcium phosphate (CaPs)-based ceramics constitute a group of biomaterials which include many different calcium phosphate phases with varying Ca to P molar ratios (from 0.5 to 2) that cause different morphologies, particle sizes, and forms, thus diverse biological performance [16]. These materials have a chemical composition and structure like the mineral phase of bone. Properly manufactured CaP ceramics have an interconnected porous structure, and they are well known for their excellent osteoconductive properties. However, they do not have proper osteoinductivity, moderating the attachment and the differentiation of osteogenic cells [17]. Porous CaP ceramics possess a compressive strength corresponding to 10–25% of the compressive strength of long bones [18]. Thus, porous CaP ceramics need more mechanical support via osteosynthesis when used in weight-bearing bone parts [19].

Owing to their unique structures, CaP ceramics facilitate the growth of bones on their surface and into their three-dimensional structure, which means osseointegration. It has been shown that osseointegration depends on the pore size of the materials. The optimal osteoconductivity of biomaterials can be achieved with pore sizes ranging from 300 to 400 μm, while the minimum pore size that is required to generate mineralized bone is considered to be 50 μm [20].

The different calcium phosphate phases can be synthesized by mixing calcium and phosphate solutions under acid or alkaline conditions. The preparation parameters and conditions used strongly determine the formed phases [21]. Only particular CaP compounds are beneficial for implantation in the body, as those with a calcium-to-phosphorus ratio lower than 1 are not fit for biological use because they are highly soluble [22]. The high osteoinductive and bone regenerative capability of these materials mainly linked to their chemical and physical properties, such as surface roughness and porosity [23,24]. It is also reported [25,26] that the presence of calcium ions and inorganic phosphate activates specific signaling routes that facilitate bone generation. The calcium phosphate powder particles can be prepared in many ways, either at low or high temperatures and in dry or wet conditions. The main technologies are summarized in Table 1.

CaP powders obtained from the above discussed methods can be processed further by integrating dopant elements, such as Sr, Zn, and Mg, or by forming composites with polymers or alternative bioceramics [27].

**Figure 2:**
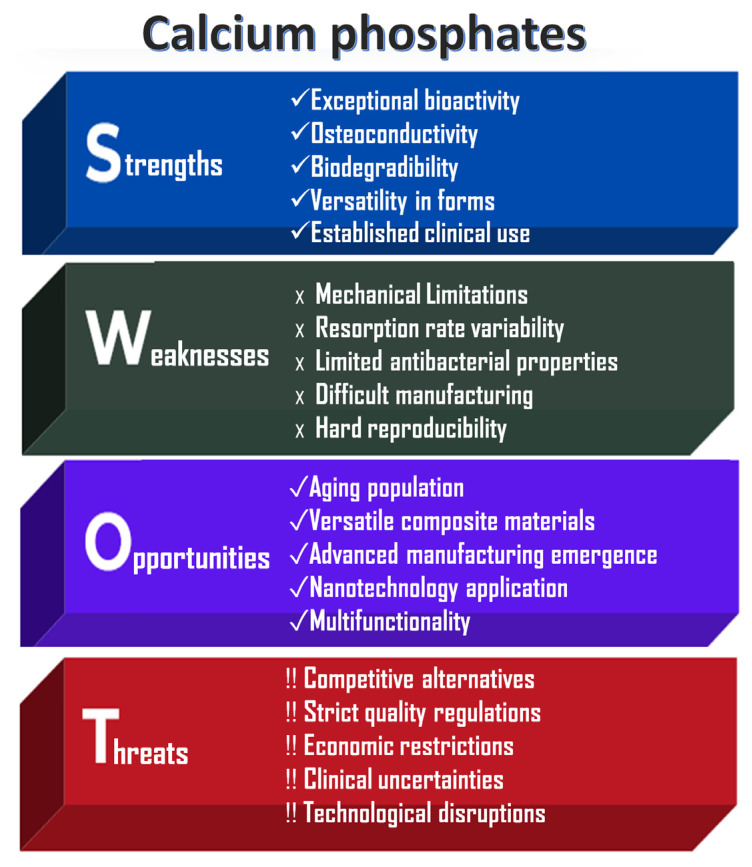
SWOT analysis of CaP-based ceramics.

It is also important to assess the practical effectiveness of these CaP powders. As shown in Figure 2, a SWOT analysis has been conducted on the applications of calcium phosphate ceramics, which underscores both their significant advantages and the challenges they present to society.

The main strengths of calcium phosphates (CaPs) lie in their exceptional chemical properties and morphology, which facilitate integration into the body through direct bonding with bones. When used as scaffolds, CaPs support bone cell migration and proliferation, thereby promoting natural bone regeneration. Their gradual degradation enables replacement by native bone; for example, β-tricalcium phosphate resorbs more rapidly than the hydroxyapatite phase [24,25,26]. Additionally, CaPs can be employed in various forms and applications, including dental, orthopedic, and craniofacial procedures.

However, several limitations affect their broader application. The inherent brittleness and limited fracture resistance of CaPs restrict their use in load-bearing regions [18,19]. Furthermore, the degradation rate may not always align with the rate of bone healing, potentially leading to premature scaffold failure or interference with tissue regeneration. Without additional surface modifications, these materials can also be prone to microbial colonization [25].

Other challenges include stringent FDA (Food and Drug Administration) and EMA (European Medicines Agency) regulatory requirements, which delay market entry and increase development costs. High production expenses and limited accessibility in resource-constrained regions further hinder widespread adoption. Moreover, there is a lack of long-term clinical studies addressing rare complications such as inflammation or inconsistent resorption behavior.

Despite these challenges, CaPs remain important biomaterials, particularly as the aging and growing population drives increased demand for bone grafts due to osteoporosis, trauma, and joint replacements. The use of advanced manufacturing technologies, such as 3D and 4D printing, offers significant potential for producing patient-specific scaffolds with complex geometries, thereby improving surgical outcomes [3].

## 3. Bioglasses

Bioglasses are a type of bioactive material that exhibit excellent biocompatibility and the ability to bond directly to bone tissue and have the function of enhancing the regeneration of bone tissues while gradually degrading [28]. The commercial composition of bioglasses is mainly an amorphous mix of oxides, such as silicon dioxide, sodium oxide, calcium oxide, and phosphorus pentoxide in different ratios. The most used composition is 45 Wt.% SiO_2_-24.5 Wt.% Na_2_O-24.5 Wt.% CaO-6 Wt.% P_2_O_5_, but many other variations, which are widely elaborated in detail in numerous reviews, are available [29,30,31].

Generally, these materials are typically amorphous silicate glasses made either by the melting method [32,33] or the sol–gel method [34]. In addition, researchers [35] compared the mechanical and physical properties of bioglass scaffolds made in two different ways. They found that the sol–gel-synthesized bioglasses are superior to melt-produced bioglasses because of their greater porosity and better bioactivity. According to the main constituent, there are three main types of bioglasses, such as silica-based glasses [36], borate-based glasses [12,37,38], and phosphate-based glasses [39,40]. Each type has its unique chemical composition and mechanical and structural properties; therefore, they can be utilized in various applications in biomedical fields. Their main applications are as bone grafts and fillers. Recent studies have demonstrated their effectiveness in promoting osteogenesis and angiogenesis [41]. The bioglasses can be prepared alkali-free, especially Na-free. Sodium is regarded as an essential component for bioactivity, since it effectively disintegrates the glass network. However, bioglasses without sodium revealed similar dissolution characteristics and bioactivity as traditional bioglasses with sodium [42,43,44]. In addition, some in vitro biocompatibility studies have proven that the bioglasses with higher Na_2_O content can be linked to cytotoxic effects [45].

**Figure 3:**
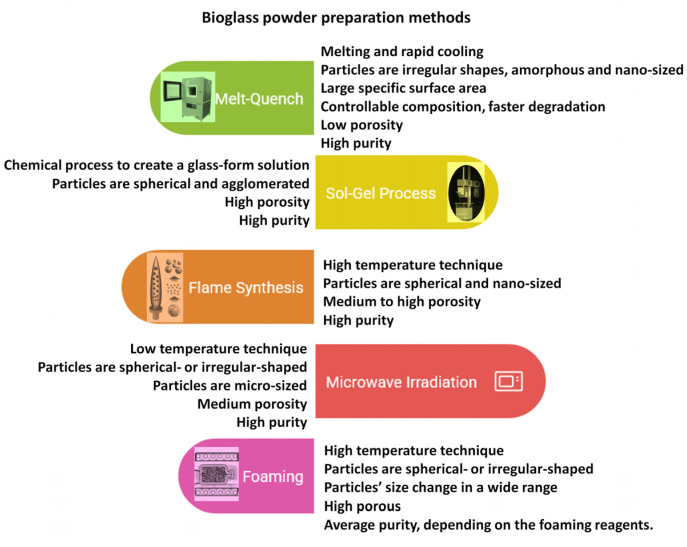
Schematic illustration of bioglass preparation methods and their properties.

It has also been investigated that the apatite formation and the degradation rate are significantly affected by the glass network porosity and the amount of phosphate. The CaO, Na_2_O, and P_2_O_5_ components are network modifiers, and they can be integrated into the original SiO_2_-CaO-Na_2_O composition to obtain a more reactive surface [46,47]. The P_2_O_5_ content as a phosphate derivative is also necessary for bioactivity [48,49]. The different types of bioglass powders can be prepared in many ways, the most widely used ones are illustrated in Figure 3 [32,42,43,44,45,46,47,48,49].

**Figure 4:**
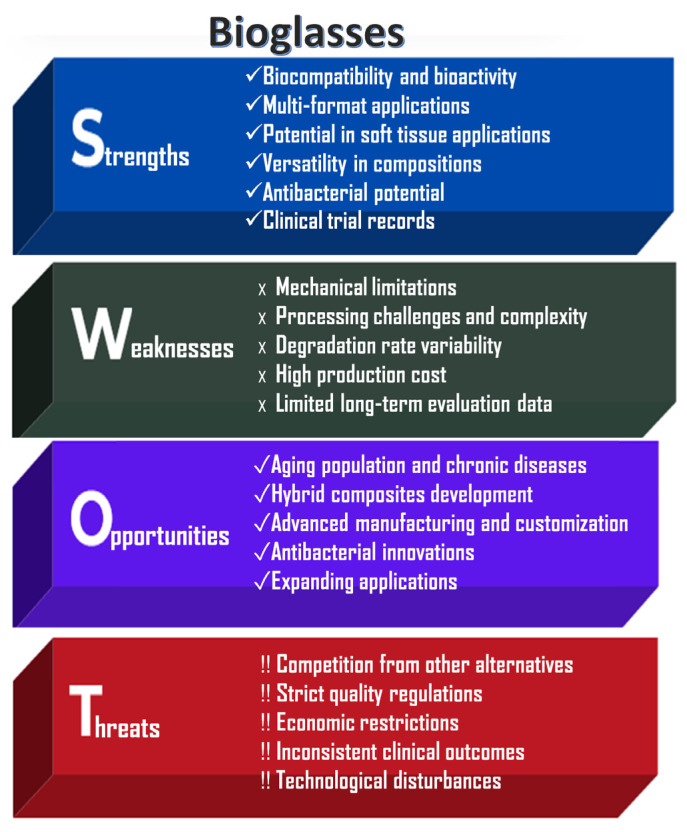
SWOT analysis of bioglass powders.

An analysis of the strengths, weaknesses, opportunities, and risks associated with bioglass powder applications, highlighting their significant societal benefits and drawbacks, is shown in Figure 4.

The key strengths of bioglass powders are their excellent bioactivity and highly versatile composition. They are capable of stimulating both osteogenesis (bone formation) and angiogenesis (blood vessel formation). In physiological fluids, a hydroxycarbonate apatite (HCA) layer can form on their surface, enabling strong chemical bonding with both bone and soft tissue [1,2,3]. Bioglasses can be engineered with tunable degradation rates and controlled ion release profiles [11]. Another significant advantage is their processability into various forms, including powders, scaffolds, coatings, fibers (e.g., bioactive glass wool for wound healing), and injectable cements. Unlike other bioceramics, bioglasses have the unique ability to bond with soft tissues, making them particularly useful in periodontal and other soft tissue applications [10].

However, bioglasses also present notable limitations. Similarly to calcium phosphate ceramics, they exhibit poor mechanical properties, including brittleness, low tensile strength, and limited fracture toughness, which restricts their use in load-bearing applications [5,6]. Large-scale production is technically challenging due to high melting temperatures and the risk of unwanted crystallization during manufacturing. Furthermore, their degradation behavior and ion release are composition-dependent, potentially leading to inconsistent or unpredictable biological responses [19].

Like other bioactive materials, bioglasses are subject to stringent regulatory requirements by agencies such as the FDA and EMA, which can delay commercialization and increase development costs. Nevertheless, ongoing innovations, particularly in composite materials, 3D printing technologies, and antimicrobial doping, are expected to expand the applicability of bioglasses in emerging fields such as personalized regenerative medicine and chronic wound care [3].

## 4. Applications of Bioactive Ceramics

The main application areas of bioactive ceramics (CaPs and bioglasses alike) are versatile. These bioceramics can be utilized in both soft and hard tissue engineering, along with their application in drug delivery mechanisms and as coatings for metal implant surfaces.

### 4.1. Bioglasses and Calcium Phosphates in Bone Tissue Engineering (BTE)

**Figure 5:**
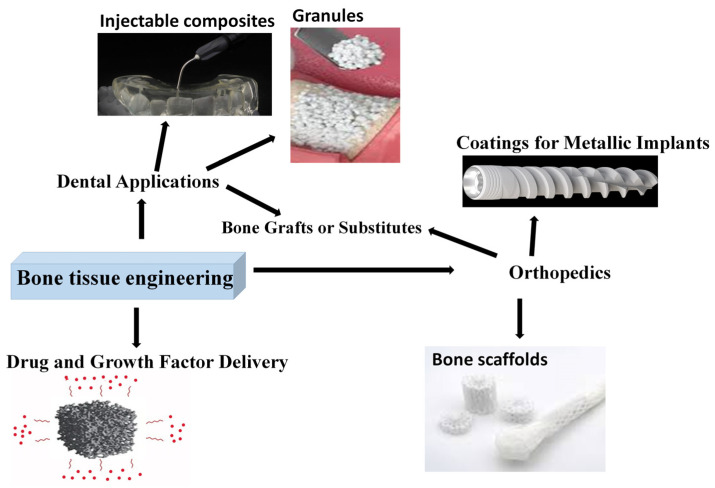
Bioactive ceramic utilization in bone tissue engineering.

The most important applications are depicted in Figure 5. As presented, the main application fields in bone tissue engineering are orthopedics, where they can be used as scaffolds, bone grafts, or coatings, and dentistry, where the most common forms are granules, bone fillers, and injectable composites. Another emerging and developing field is drug delivery matrices.

#### 4.1.1. Scaffold Materials and Bone Substitutes

In clinical practice, bone irregularities are prevalent and impose a considerable burden on the families of patients, as well as on society and healthcare infrastructures. Scaffolds made from calcium phosphate with complex nano- and micro-level structures have proven to be highly effective in facilitating bone regeneration. These materials can be fabricated into porous scaffolds that support cell attachment, proliferation, and differentiation [50,51,52,53].

Calcium phosphate cements (CPCs), as unique forms of CaPs, are synthetic, self-assembling bone substitute materials. The main benefit of CPCs is that they are injectable, moldable, and hardened in situ, so the contact between the tissue and the implant will be optimal, even when the defect dimensions are irregular [49]. Calcium phosphate scaffolds provide stable properties and allow the control of porosity and biocompatibility. The pore size of the scaffold improves revascularization and bone remodeling, enabling the ingrowth of cells and proteins and enhancing biocompatibility, making them suitable for implant use [54].

**Figure 6:**
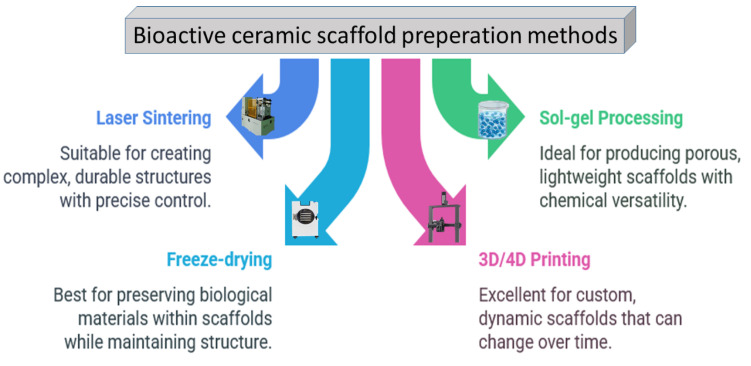
The most utilized ceramic scaffold preparation methods so far.

As depicted in Figure 6, a range of approaches is used to fabricate porous ceramic scaffolds, and each method has a unique impact on the mechanical properties and the suitability of the end products for specific applications [55,56].

However, numerous other methods and additives can be utilized to modify the porous characteristics of ceramic scaffolds. For example, adding a pore-forming agent [57], applying foam technology [58], or extrusion molding method [59]. Nonetheless, the connectivity of pores plays a crucial role in determining the biological performances, and at the same time, a three-dimensional arrangement of connected pores has been found to be more susceptible to a decrease in the mechanical properties of ceramic materials [60,61].

Bioglasses and CaPs have a similar role in bone tissue engineering thanks to their ability to directly adhere to bones. They function effectively as bone grafts, fillers, and restorative materials, promoting rapid integration and regeneration. Preparation methods like melt-quenching and sol–gel facilitate the development of porous structures with adjustable characteristics. Specifically, the ideal method for bioactive glass preparation is the sol–gel technique [62]. It helps to customize the properties of materials for bone tissue regeneration, because they can degrade at a controlled rate, transform into bone-like material, and connect well with tissues while promoting bone-forming cell growth. Adding bioactive elements or drugs into these bioglasses can further enhance their ability to heal bone and prevent infections. One of the most ideal structures for scaffold materials is the mesoporous system [63,64] that can be prepared by 3D printing as hierarchically porous scaffolds. However, there are still existing challenges regarding their applicability due to insufficient mechanical strength, especially for load-bearing bones [31,65].

The mechanical characteristics of bioglass and CaP-based scaffolds have been compared to natural bone and presented in Table 2 [5,6,7,19,40,57,66,67].

While dense bioceramics (bioglasses and CaPs) excel in compressive strength and hardness, their brittleness and low fracture toughness make them unsuitable for dynamic load-bearing applications. On the other hand, natural bone’s composite structure provides a unique balance of strength, toughness, and self-repair. Scaffold materials prioritize bioactivity and controlled degradation over mechanical performance, aligning with their role in temporary bone regeneration.

Another key challenge in the field of bone tissue engineering is the imitation of the extracellular matrix’s composition. Scaffolds derived from bioglass particles and hydroxyapatite feature a naturally porous architecture that enhances cell attachment and development. In addition, it can enable vascularity, migration of nutrients, and metabolic by-products. The unique structures of these materials ensure better adherence of osteoblast cells, promoting their faster proliferation, differentiation, and mineralization into bone [68]. This represented a significant advancement in the development and optimization of different bone filler materials [69].

#### 4.1.2. Bioactive Ceramic–Polymer Composites

In tissue engineering, another interesting area is the development of composite materials that combine bioglasses and calcium phosphates with different biopolymers to enhance their suitability and adjustability.

**Figure 7:**
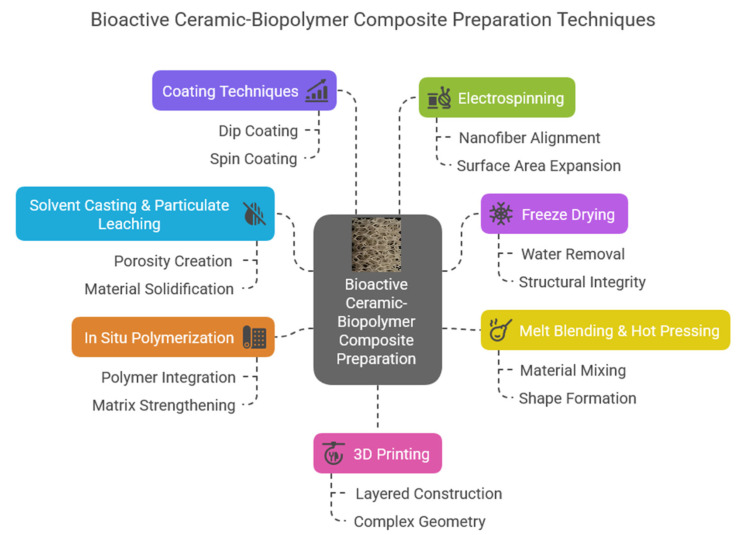
Bioactive ceramic and biopolymer composite preparation possibilities and their forms.

As Figure 7 shows, composite scaffolds can be prepared in many ways. Each has its advantages and disadvantages. These unique composite materials (both bioglasses and calcium phosphate derivatives) can be prepared as solid scaffolds, injectable composite hydrogels, fiber mats, thin films, or even matrices [70]. Among the various types of these composites, the injectable form is exceptionally attractive and useful [71,72,73]. The most commonly used biopolymers to form these composites can be classified into two main categories. One is the natural biopolymers, such as cellulose, cellulose acetate, alginates, chitosan, collagen, gelatin, and the other is the synthetic polymers like polylactic acid (PLA), polyvinyl pyrrolidone (PVP), polycaprolactone (PCL), and polyethylene glycol (PEG). A wide array of materials has been explored for the fabrication of these polymer-based scaffolds, each offering unique properties and functionalities tailored to specific applications. Chiu et al. [74] prepared injectable implants in the form of calcium sulfate and self-setting calcium phosphate composite. The developed injectable pastes were easy to handle, had excellent biocompatibility, and sufficient mechanical properties. These pastes also had great potential in minimally invasive surgery, and they can be utilized to treat maxillofacial defects or even in reconstructive rhinoplasty. In other interesting work, Cai et al. [75] prepared injectable chitosan oligosaccharide (COS) and bovine-derived hydroxyapatite (BHAp) composites. The intended use of this material was as a dental pulp cap. The developed biocomposite was slightly soluble in physiological solutions; however, the solubility rate can be changed by applying certain cross-linking agents. Regarding bone regeneration, a promising composite can also be a dextran-based injectable hydrogel [76] which can be utilized as scaffolds and drug-release systems simultaneously.

The alginate-, chitosan-, collagen-, and gelatine-based composites are similarly ideal choices to prepare injectable materials. For example, Xu et al. [77] prepared bioactive glass containing sodium alginate hydrogel that had immunomodulatory and angiogenic properties to heal tendon tissues. According to their results of the biomechanical tests, the BG/SA hydrogel noticeably enhanced the load, failure stress, and tensile modulus of the repaired tendon. Therefore, applying injectable BG/SA hydrogel can be a novel and promising therapeutic approach to heal Achilles tendons along with surgical intervention. On the other hand, in a recent study, Estevez et al. [78] developed calcium phosphate containing sodium alginate and gelatin hydrogels as bone replacement injectable composites. They proved that the developed hydrogel composites had mineralization ability with slight antimicrobial properties and a slow-to-moderate degradation rate. Wu et al. [79] recently reported their work on the preparation of porous composites microspheres based on hydroxyapatite (HAp), di-calcium phosphate dihydrate (DCPD), and chitosan. They prepared the composites via the hydrothermal method, in which chitosan had an important role as a chelating agent to promote the calcium phosphate particles’ growth. They found that the formed microspheres comprising Ca_2_P_2_O_7_, β-TCP, and HAp can be utilized as injectable bone graft materials. Chitosan is also an important matrix for injectable hydrogel preparation. Ciotek et al. [80] examined chitosan and sodium hyaluronate hydrogels with bioglass particle addition under different conditions and found that these bioglass and polysaccharide hydrogels were microstructurally stable and bioactive. Besides the above-mentioned works, numerous other researchers are exploring these kinds of important injectable composite materials [81,82,83,84].

In the form of solid and stable scaffold materials, these novel composites can be utilized in cases of larger bone deficiency as filler materials. In this case, the main preparation methods are molding and additive manufacturing, such as 3D or 4D printing [85,86,87].

For instance, PLA polymer with embedded calcium phosphate particles has been evaluated for its bioactivity [88]. In vitro studies have shown that human mesenchymal stromal cells (hMSCs) proliferate on these composites, indicating potential for tissue engineering applications. In other research, Zhang et al. [89] investigated the effect of different amounts (5%, 10%, and 20% in wt%) of 63s bioglass on the properties of bioglass-PCL composites as scaffolds. They shaped the scaffold with 3D printing. The results revealed that the performance of the composites improved by increasing the 63s BG content. However, despite their advancements, they did not successfully reproduce the complex mechanical characteristics of cortical bone, which limits their application in load-bearing orthopedic settings, but they are appropriate for addressing minor bone or cartilage repairs. Rajzer et al. [90] prepared bioglass and zinc-doped bioglass containing PCL composite filaments by injection molding. They studied the mechanical and biological properties of the composites. In vitro mineralization studies revealed apatite formation on the surface of bioglass-added scaffolds after 7 days of immersion in SBF; however, in the case of Zn-doped bioglass, slower apatite formation was observed. An interesting work [91] reported a 3D preparation of biphasic calcium phosphate-PCL scaffolds with high and interconnected porosity as well as chemical degradability. They found that the PCL content improved the strength and toughness of composites, that is important to withstand mechanical loading in bone tissue engineering.

Hajiali et al. [92] compiled a thorough review paper on the PCL-bioglass and PCL-CP composites and studied their mechanical and biological performances as well as their biodegradability. In their paper, they focused on the preparation methods of scaffolds with ideal mechanical and structural properties; however, they did not discuss or draw any conclusion about the differences between the bioglass and CP containing PCL matrices. They concluded that the conventional preparation methods cannot produce scaffolds with controllable pore sizes, interconnectivity and reliable internal as well as external structures. Thus, their mechanical characteristics do not meet the requirements. However, the most ideal preparation method, according to their paper, is the FDM 3D printing process, which deposits melted composite filaments over a build platform layer by layer. This provides excellent mechanical and biological properties for the entire scaffold.

A recent study [93] reported that the PCL polymer has no inherent antibacterial characteristic; therefore, it can be subject to bacterial attachment and biofilm generation. They tried to make the composite bactericidal by CaP nanoparticle incorporation into the polymer matrix. They stated that the flake-like morphology of the prepared CaP particles could disrupt bacteria’s membranes, thus impeding bacterial growth.

Another paper [94] described the preparation of novel, three-layer calcium phosphate/polycaprolactone scaffolds. These scaffolds had a graded increased porosity fraction from the inner to the outer layer. In addition, they were bioresorbable at a gradual rate. The scaffolds are composed of a dense hydroxyapatite (HAp)/β-tricalcium phosphate inner layer, a macroporous HAp/β-TCP intermediate layer, and lastly a macroporous PCL/(HA/β-TCP) outer layer. The preparation methods were gel-casting for the inner layers, while the outer composite layer was formed by a solvent casting and particle leaching technique. They carried out in vitro dissolution tests in TRIS solution, and the results showed that the dissolution happened in a differentiated mechanism within the different layers. They concluded that a targeted multi-functionality can be reached with this layered structure.

In earlier research, Ródenas-Rochina et al. [95] compared the properties of hydroxyapatite and bioglass (BG) nanoparticles in a polycaprolactone composite scaffold. They found that pre-osteoblast cells proliferated well on all scaffolds. However, in their study, the addition of bioactive particles did not cause noticeably more positive effects on cell differentiation than that of a pure PCL scaffold.

Besides the widely investigated HAp and TCP calcium phosphate phases, another promising filler material can be the octacalcium phosphate (OCP), which has been combined with various polymers to create composites for tissue regeneration. For example, gelatin–OCP composites have been studied for their ability to support osteointegration due to their inherent porosity. In vivo preclinical studies have demonstrated that these composites can regenerate substantial amounts of bone within months of implantation, suggesting potential applications in both bone and soft tissue engineering [96].

### 4.2. Bioglasses and Calcium Phosphate Derivatives in Soft Tissue Engineering (STE)

While primarily associated with hard tissue repair, bioglasses and calcium phosphate derivatives can also find applications in soft tissue engineering. Unfortunately, their use in soft tissue regeneration is less common and requires further research. However, they can be prepared in ideally porous structures that can modulate cell behavior. This makes them suitable for cartilage, tendon, and ligament repair [97,98,99]. Techniques like freeze-drying, hydrogel incorporation, and electrospinning are employed to tailor these materials for soft tissue applications. Hence, nanostructured calcium phosphate is an essential biomaterial for hard tissue repair, but also has potential in some soft tissues, as reported by a recent review [10].

**Figure 8:**
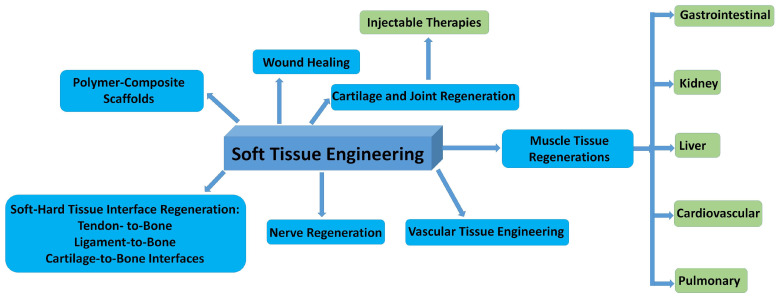
Schematic illustration of the most important fields of bioceramic–biopolymer composites use in STE.

In Figure 8, the possible fields of applications in soft tissue repair are summarized.

The main goal in soft tissue engineering is to repair and restore damaged or diseased tissues and remove the disadvantageous properties of both allografts and autografts. These materials can be prepared with optimized flexibility, biocompatibility, and biodegradability. These novel materials can initiate neovascularization, which is important for host tissue integration with the implanted structure [100]. Some reports describe their ability to aid the healing process of acute and chronic wounds [101,102,103,104]. In addition, bioglasses and calcium phosphate derivatives can be used to regenerate heart and lung tissues that are commonly known for their poor renewal capacity, as well as to repair peripheral nerve and musculoskeletal tissues [10,100,105,106,107,108]

Extensive research revealed that the incorporation of ceramic nanoparticles significantly improves mechanical strength and biological characteristics and alters the degradation kinetics of polymers. In addition, it can noticeably improve flexibility, which is crucial for soft tissue applications [109]. Furthermore, bioglasses have also proven their capacity to promote skin healing by improving blood vessel formation and collagen production during the proliferation phase, along with beneficial impacts on all other critical stages of the wound healing process [38].

It is noteworthy that since bioactive calcium phosphate-based ceramics are primarily used in association with bones, the number of reported data utilizing them in the field of wound healing is rather limited. For example, Eliaz et al. [110] discussed in a review paper that calcium phosphates have also shown potential as wound dressings since the cellular reaction is different for hydrophilic and hydrophobic implants, particularly in the initial stages of wound healing. CaPs can provide sufficient hydrophilicity to the surfaces with higher surface energy, resulting in faster cell activation and differentiation than those with lower surface energy.

Wang et al. [111] also mentioned in their mini review that traditional phosphate-based (Ca_3_(PO_4_)_2_, Ca_5_(PO_4_)_3_(OH)) and silicate-based (CaSiO_3_ and MgSiO_3_) powders can be changed into black bioceramics by the addition of magnesium and thermal reduction causing structural defects and oxygen vacancies. These black bioceramics could efficiently and controllably release Ca, Si, and Mg ions that were reportedly beneficial in the cases of skin-tumor-bearing mice [112]. Furthermore, black bioceramic-based materials have the potential to substantially boost the healing process of skin injuries in mice through improved tissue regeneration. In another review [103], calcium phosphate ceramics were also mentioned regarding their applications in wound treatment.

### 4.3. Bioglasses and Calcium Phosphate Derivatives as Drug Delivery Matrices

Nanostructured CaPs and mesoporous bioglasses with large surface areas are suitable for drug/gene delivery systems. These matrices are suitable architectures for incorporating and releasing drugs, growth factors, therapeutic agents, and even genes in a controlled way. Common preparation methods can be co-precipitation [113] and layer-by-layer assembly [114,115], which can control release kinetics and enhance efficacy. In addition, they have a moderate and gradual degradation rate in different biological conditions, as well as a large surface area. The CaP derivatives dissolve faster at a slightly acidic pH, which enables a controlled drug release into cells [116]. It is also discussed that the drug delivery ability of bioceramics (CaPs) is dependent on their crystalline rate, relative surface area, microstructure, micro- and nano-morphology, as well as particle sizes and forms [117]. Structures with low crystallinity or amorphous phases with high surface areas can incorporate more drugs and have lower release rates compared to crystalline structures, such as HAp [118]. The higher the solubility of the CaP phases, the faster the drug release rate [119]. For example, Uskoković et al. [120] reported that spheroid CaP particles had more effective drug loading and release capacity than flaky, brick-like, or elongated orthogonal-shaped particles. A large number of therapeutic agents can be delivered by bioactive ceramic nanoparticles, including antibiotics, anti-inflammatory drugs, and growth factors for bone healing [117].

Bioglasses can also serve as versatile platforms for drug delivery, capable of releasing therapeutic agents in a controlled manner. Their composition can be adjusted to achieve desired release profiles, utilizing techniques such as ion exchange and nanoparticle embedding. Their porous structure is an ideal matrix for drug inclusion, delivery, and controlled release [121]. The other unique structure of bioglasses is the mesoporous (MBGs) [122]. The mesoporous channels in bioglasses improve textural properties and bioactivity compared to sol–gel bioglasses, while their large pore volume allows simultaneous loading of drugs, growth factors, or other bioactive molecules [123]. Additionally, the silanol groups at the surface of MBGs enable further modification strategies with different functional groups for the controlled release of therapeutic compounds [124,125].

Yao et al. [126] developed a mesoporous bioactive glass (MBG) modified with alginate. They reported that by mixing MBGs with the polymer, the otherwise immediate and fast release of incorporated drug molecules can be better controlled. They have used simvastatin (SIM) as a model drug. Generally, any antibiotic incorporation is useful to prevent or cure infections at the implanted sites. For example, Huang et al. [127] prepared boron-doped bioactive glass (BG) scaffolds using the sol–gel method. Boron content aimed to enhance the bioactivity. The microstructural analyses showed mesoporous structures. They further revealed that the boron-doped BG had nano-sized pores and rougher microstructures than those of pure bioglasses, which is beneficial for drug delivery. In their case, the incorporated drug was teicoplanin. Teicoplanin and vancomycin are the last generation of antibiotics against infections caused mainly by Gram-positive bacteria. Teicoplanin is a better choice for use since it has a lower toxicity and longer half-life. According to the drug-release tests, the release rate gradually slowed down during immersion for 7 days.

The other important agents are the different growth factors, such as bone morphogenetic proteins (BMPs), platelet-derived growth factor (PDGF), vascular endothelial growth factor (VEGF), and transforming growth factor-beta (TGF-β) are all critical factors in bone healing [128]. Hence, the addition of growth factors can further enhance the osteogenic ability of bioactive ceramics. For instance, BMP-2 connects with CaPs through the functional groups, such as -OH, -NH2, and -COO [129]. However, it is important to denaturalize the protein during its incorporation into the ceramic matrix, otherwise, it would lose its main functionality. The addition of different genes into the system also promotes and facilitates bone regeneration [130].

Bioactive element doping also alters the drug-encapsulation capacity of bioglasses. In a recent work [131], therapeutic element-added mesoporous bioactive glass nanoparticles (MBGNs) were evaluated as unique multifunctional systems. These ions were strontium and magnesium (SrMg-MBGNs). According to their results, the Sr and Mg-doping increased pore volume, thus higher solubility rate. In addition, the original mesoporous structure changed from worm-like to radial–dendritic, which caused a little faster drug release compared to undoped MBGNs. They used ibuprofen for the drug-release experiments. The experiments showed that the drug loading capacity slightly decreased while the release rate increased due to the open dendritic pores at the external surface, which resulted in accelerated ibuprofen emission. Additionally, several studies have been actively carried out to improve the efficacy of bioactive ceramics in combination with various healing agents [132,133,134,135,136,137,138]. The most important preparation techniques are described briefly in Table 3.

### 4.4. Coatings on Metallic Implants to Improve Osseointegration

So far, an enormous amount of work has been undertaken to create coatings that improve the biocompatibility of commercially used metallic implant materials, such as titanium, Ti6Al4V, CoCrMo, and stainless steel, while also aiding in the prevention of implant-assisted infections. Coating materials such as bioglasses and phosphate-based ceramics are favored for bone implants due to their ability to promote integration with host tissues and enhance biological functionality, as it is widely discussed in many review papers [139,140,141,142]. At the junction of the bone implant and surrounding bone tissue, an apatite-like layer develops that simulates natural bone, due to the excellent bioactivity of these coatings. These layers form a robust and direct bond, ensuring the long-lasting stability of the implant within the human body [143]. In the research work of Nezami et al. [144], 58S tape bioglass powders were prepared via the sol–gel technique with subsequent calcination. The coatings were deposited electrophoretically. They studied the dissolution characteristics of the coatings by immersion tests. The results revealed excellent bioactivity and corrosion resistance of the coatings. While Zanca et al. [145] prepared CaP-bioglass composite coatings onto stainless steel substrates by the galvanic co-deposition of calcium phosphate and bioglass particles. According to their results, these coatings also had excellent biocompatibility and a low corrosion rate. In another recent paper, Farjam et al. [146] deposited CaP coatings onto polycarbonate–urethane (PCU) foils. In this case, the PCU samples were immersed in supersaturated SBFs, which resulted in a semi-crystalline CaP coating formation on the flexible foil over time. Such prepared CaP coatings were stable and intact when the foil was bent. The in vitro cell viability tests revealed that these coatings did not affect the cell viability and proliferation compared to the uncoated PCU substrate. Moreover, the CaP coatings promoted cell-mediated calcium deposition.

In another work [147], the preparation of double-layered porous CaP coatings onto Ti6Al4V was reported. The inner coating was deposited by plasma electrolytic oxidation (PEO), while the outer one was by radio frequency magnetron sputtering (RFMS). They used different CaP targets, such as β-tricalcium phosphate, hydroxyapatite, Mg-added β-tricalcium phosphate, Mg-added hydroxyapatite, Sr-added β-tricalcium phosphate, and Sr-substituted hydroxyapatite. They revealed that the RFMS treatment of PEO inner coating caused multileveled roughness, increased the Ca/P ratio and Young’s modulus, and helped to alter the crystallinity of the coating. Duta et al. [148], on the other hand, investigated the differences between the synthetic and naturally derived hydroxyapatite coatings. They reported that natural HAps contain a wide variety of trace elements (such as Na, Mg, Sr, and K) compared to synthetic ones. Therefore, they can have a more dynamic connection with the surrounding biological area.

**Figure 9:**
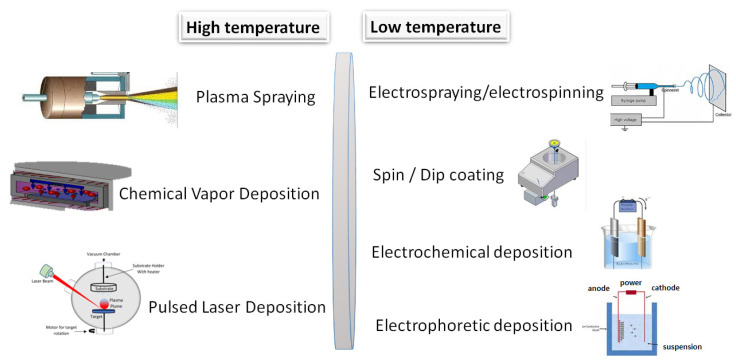
Schematic illustration of the most utilized coating techniques.

Gao et al. [149] applied CaP coatings onto magnesium alloy to enhance its biocompatibility and to decelerate the degradation rate of bare magnesium implant material. The most common methods are depicted in Figure 9 and categorized as high and low temperature methods.

These deposition techniques and their main characteristics are discussed in detail in many review papers [150].

Table 4 demonstrates the main characteristics (strengths and deficiencies) of coatings prepared by the described methods [150]. Even though these bioactive coatings have many benefits, their performance in load-bearing applications is constrained by several critical limitations, such as mechanical (for example, brittleness, insufficient fracture toughness, and cracking) as well as poor adhesion to substrates. In addition, there are degradation challenges, namely, uncontrollable dissolution rates. Bone regeneration requires coatings to degrade at a rate matching new bone formation (~3–6 months). However, HAp coatings often last for years, interfering with bone remodeling. On the other hand, bioglass coatings may degrade too quickly (<3 months), leaving the implant surface exposed before osseointegration is complete [151].

### 4.5. Effects of Bioactive Element Doping of Bioglasses and CaP Derivatives

It has been extensively demonstrated that the mechanical, chemical, and morphological properties of both bioglasses and calcium phosphate-based bioceramics can be modified and optimized through the incorporation of various bioactive minerals or metallic ions. Commonly used dopants include elements such as silicon (Si), strontium (Sr), magnesium (Mg), zinc (Zn), iron (Fe), copper (Cu), cobalt (Co), fluoride (F), and silver (Ag), introduced in the form of either organic or inorganic salts [152,153,154]. These dopants have been shown to influence the crystal structure, nano- and micro-scale morphology, and surface topography of bioceramic substrates, thereby impacting their biological performance. Their effects have been extensively discussed and evaluated in the scientific literature [155,156,157,158,159,160].

In this context, the most commonly used and investigated substitute elements and their biological functions are further examined. Theoretically, these doping elements become incorporated within the interconnected pores of scaffolds and coatings. Upon implantation, surrounding body fluids infiltrate the porous structure, gradually dissolving the incorporated ions depending on their individual solubility characteristics. However, in the dynamic and fluctuating physiological environment, these dissolution rates may vary and, in some cases, accelerate.

**Figure 10:**
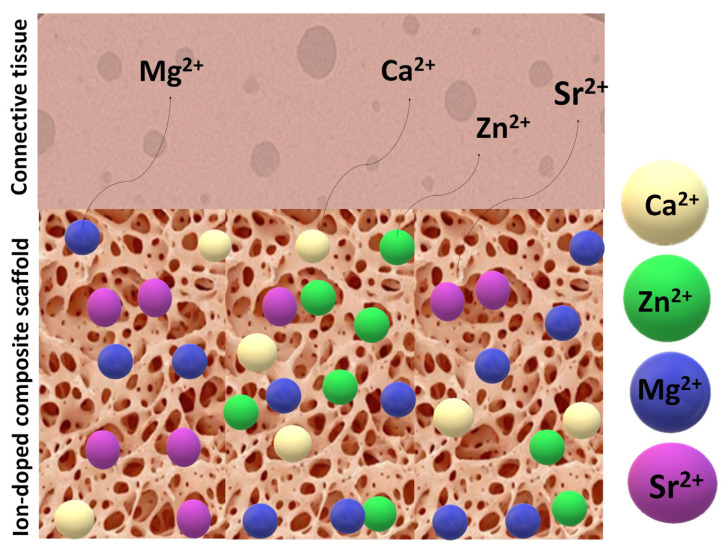
Illustration of an ion-doped porous scaffold in contact with tissues and the doping ions’ dissolution pathway.

Figure 10 illustrates a porous scaffold material infused with bioactive ions interacting with adjacent tissue, alongside a proposed mechanism for ion release and leaching.

In Table 5, the biologically most active doping elements are listed (along with their roles) that can enhance the effectiveness and bioactivity of the base bioglasses and CaP ceramics or composites [156,157,158,159,160].

We further analyzed the doping components’ influence on a molecular scale and investigated their interactions with tissue or bone cells that can enhance and accelerate the healing process (Table 6) [156,157,158,159,160].

## 5. Biodegradability and Clinical Evaluations of the Different Bioactive Ceramics and Bioglasses, as Well as Their Composites

Generally, both bioglasses and calcium phosphate (CaP) derivatives represent a unique class of bioactive materials capable of interacting with body fluids to form a biologically active hydroxycarbonate apatite (CHAp) layer on their surfaces. This reaction not only facilitates bonding with bone but also promotes new bone formation. The biodegradability of these materials is strongly influenced by their ionic dissolution mechanisms and rates [161,162,163,164,165,166,167], their chemical composition [168,169], and the surrounding environmental pH [170]. For example, Gharbi et al. [171] reported that high boron concentrations in bioglasses significantly increase their degradation rate. The resulting release of bioactive ions can enhance osteogenesis and angiogenesis and, in some cases, confer antibacterial properties.

Surface topography and micromorphology are also critical factors in the biological performance of both bioactive ceramics and bioglasses. Studies have shown that a rough and porous surface greatly enhances cell adhesion [172,173]. Such surface structures also facilitate controlled drug release and improved cellular interactions, rendering these materials highly suitable for a variety of medical applications.

Understanding the influence of the network structure of bioactive ceramics on the solubility of bioglasses is crucial for designing materials with tailored biological functions. A well-established correlation exists between the structural and morphological properties of bioactive ceramics and bioglasses and their biological performance [174,175].

Theoretically, the degradation of bioglass scaffolds begins with an ion exchange upon contact with body fluids [176]. In silicate-based bioglasses, for instance, sodium and calcium ions are leached out and replaced by hydrogen ions, increasing the local pH and leading to the formation of a silica-rich layer. This is followed by the precipitation of calcium and phosphate ions, forming an amorphous calcium phosphate layer, which subsequently crystallizes into CHAp [177]. Borate-based bioglasses exhibit a less dense network structure, resulting in faster degradation and more complete conversion to CHAp [178]. Phosphate-based bioglasses, on the other hand, are known for their even higher solubility, often undergoing complete dissolution under physiological conditions. An appropriate degradation rate is vital to ensure synchronization with new tissue formation, thus promoting optimal healing outcomes [179].

Conversely, the biodegradation of CaP ceramics predominantly occurs through a combination of dissolution and cell-mediated resorption [180]. As with bioglasses, the degradation rate of CaPs is a critical parameter in tissue engineering, ideally matching the rate of tissue regeneration. In aqueous or physiological environments, CaP materials dissolve gradually, releasing calcium and phosphate ions. Hydroxyapatite (HAp), for instance, is highly stable and exhibits slow degradation, making it ideal for load-bearing applications where longevity is essential. β-Tricalcium phosphate (β-TCP) possesses relatively higher solubility than HAp, releasing bioactive ions more readily and thus supporting osteogenesis. Another commonly used CaP phase is biphasic calcium phosphate (DCP or monetite), which offers a more adjustable degradation rate, allowing synchronization with bone regeneration. The degradation rate is further influenced by factors such as crystallinity, particle size, porosity, and the presence of ionic dopants. As degradation proceeds, the released ions can stimulate osteoblast proliferation and differentiation and enhance angiogenesis at the defect site [181].

The composition and microstructure of bioactive ceramics also significantly affect their degradability. Studies indicate that degradation rates can be fine-tuned to align with tissue regeneration needs [28]. Over the past decades, extensive preclinical and clinical research has demonstrated the effectiveness of bioglasses, such as the silicate-based 45S5 Bioglass^®^, the S53P4 formulation, borate-based variants, as well as calcium phosphate derivatives in various clinical applications [182,183,184].

Several studies have shown that combining bioglasses with local autografts leads to improved clinical outcomes without notable side effects, infections, or immune reactions [185]. For instance, Cottrill et al. [186] conducted a systematic review and meta-analysis on the use of bioglasses in spinal fusion, concluding that these materials offer promising potential as autograft extenders. Similarly, Van Vugt et al. [187] performed a mid-term clinical evaluation of S53P4 bioactive glass in the treatment of chronic long bone osteomyelitis. In this study involving 78 patients, infection was eliminated in 85% of cases, and 89% remained infection-free after one year. These outcomes support the use of S53P4 in one-stage osteomyelitis treatments.

Further clinical investigations have confirmed the effectiveness of S53P4 bioglass in osteomyelitis treatment [188]. In one such study involving 50 patients, 70.3% recovered within six months, and 83.3% showed no signs of infection after twelve months without the use of antibiotics—an effect attributed to the inherent antibacterial properties of the material. S53P4 has also been successfully applied in craniofacial reconstruction, with its sustained release of alkali ions increasing pH and eradicating infection, while concurrently degrading and supporting bone formation. Long-term follow-up studies report high fusion rates and favorable functional outcomes [189,190,191].

Additionally, composites integrating bioglass and fiber reinforcements have shown promise in maxillofacial and cranial surgery applications, demonstrating success in repairing various bone defects [182,192]. Animal model studies have also confirmed that certain bioglass derivatives enhance bone regeneration and improve implant osseointegration [193]. For example, in vivo studies using small animals (e.g., rats and rabbits) have demonstrated that 45S5 Bioglass^®^ gradually degrades and is replaced by newly formed bone within a few months [194,195,196]. Borate-based bioglasses have been associated with accelerated bone healing due to their higher degradation rates, although potential toxicity is managed by regulating ion release in dynamic physiological conditions [12,197]. Phosphate-based bioglasses, due to their compositional similarity to bone minerals, also show enhanced osteogenic potential [198].

Several animal studies have reported complete degradation of bioglass scaffolds within 3–6 months, accompanied by the formation of well-vascularized mature bone tissue and minimal inflammatory response [199]. Clinical trials utilizing 45S5 Bioglass^®^ (marketed as PerioGlas^®^ or BioGran^®^) have shown success in periodontal therapy [200], with improvements in probing depth and bone defect filling. Long-term data indicate that bioglass facilitates not only bone formation but also regeneration of the periodontal ligament and cementum, resulting in predictable clinical outcomes [201].

Moreover, emerging clinical evidence supports the application of borate-based bioglasses in wound healing and soft tissue regeneration. Products such as MIRRAGEN^®^ have been approved for treating chronic wounds, highlighting the material’s versatility [202]. Clinical studies further confirm the efficacy of bioglass-based treatments in enhancing bone regeneration with minimal long-term adverse effects [203].

Calcium phosphate derivatives have also been thoroughly investigated as bone substitutes. Their tunable degradation rate, governed by phase composition, crystallinity, porosity, and doping, allows for gradual replacement by newly formed bone [204,205,206,207]. Extensive in vitro and in vivo studies, along with clinical trials, have demonstrated the positive biological responses elicited by CaP materials in bone repair. In vitro evaluations focused on cell viability, adhesion, and cytocompatibility, highlighting the influence of surface morphology and crystallinity on osteoclast-mediated resorption and osteoblast proliferation [208,209,210,211]. Osteoblasts cultured on HAp and β-TCP surfaces exhibit high levels of viability, adhesion, and expression of osteogenic markers such as alkaline phosphatase, osteocalcin, and Runx2, which support tissue mineralization [212]. Similar ion-mediated mechanisms operate in bioglasses, where ionic dissolution promotes early tissue mineralization [213].

Simulated body fluid (SBF) immersion tests are frequently used to evaluate CaP bioactivity. These studies reveal that HAp and β-TCP can facilitate apatite layer deposition on their surfaces within 7–14 days, suggesting their strong bone-bonding potential [214,215,216]. In vivo assessments using various animal models (e.g., rabbits, dogs, and rodents) further confirm the performance of CaP materials in healing critical-sized bone defects [183,217,218,219,220]. HAp’s slow degradation rate makes it suitable for long-term scaffolding, with histological analyses demonstrating continuous bone remodeling beyond six months [184,221,222]. Conversely, β-TCP undergoes complete resorption and bone replacement within 3–6 months in non-load-bearing sites [223,224,225,226]. Monetite (DCP), with its balanced resorption rate, has shown favorable outcomes in animal models, particularly when used in biphasic forms (e.g., 60/40 HAp/β-TCP), where scaffolds were largely resorbed and replaced by vascularized bone within 12 weeks [227,228,229,230].

In dentistry, HAp and DCP have been employed as graft materials and coatings for implants, with studies reporting enhanced alveolar bone regeneration, improved implant stability, and superior long-term outcomes compared to autografts or allografts [231]. In orthopedic applications, CaP ceramics are widely used for fracture repair and reconstruction. Clinical data demonstrate that β-TCP and DCP granules support rapid bone healing and reduce implant loosening risk. Trials show high success rates in periodontal and long-bone defect treatments, with β-TCP yielding comparable results to autografts over 1–2 years [232,233].

In summary, clinical assessments have confirmed the efficacy of various calcium phosphate-based scaffolds in promoting bone regeneration in critical-sized defects [234,235]. Recent in vivo and clinical studies further support that the incorporation of bioactive elements into CaP scaffolds enhances biological performance, accelerates healing, and reduces infection rates. For instance, strontium-doped CaPs have demonstrated improved mechanical strength and bone formation both in vitro and in vivo [236,237,238,239].

Table 7 and Table 8 provide a succinct summary of the bioactive glass, calcium phosphate ceramics, and their composites that are currently on the market and in use.

The specific characteristics and possible clinical applications have been extensively reviewed and described in other reviews [194,252].

Similarly, there are numerous commercial CaP-based ceramic compounds, some of which are demonstrated in Table 8.

**Table 8:** Commercially available calcium phosphate-based ceramic products and their main applications.

Brand name	Description	Application	Supplier
Eurobone^®^ 2	Synthetic bone substitute, paste granules with a composition of 75% HA/25% β-TCP.	Orthopedic (Non-load-bearing applications)	[245]
Neobone^®^	Synthetic bone substitute, putty, is an injectable synthetic bone substitute used to fill defects without mechanical strength.	Orthopedics Dentistry Bone grafts Bone substitutes	
Ostibone^®^			
NANOGEL^®^	Synthetic bone substitute. Absorbable bone void filler that provides support for bone ingrowth. Hydroxyapatite particles between 100 nm and 200 nm.	Orthopedic, Dental	
SKUHEAL™ SM-C	Biomimetic mineralized collagen synthetic bone graft material: It contains type I collagen and nano-hydroxyapatite.	Orthopedics Skull surgery Maxillofacial surgery	
HydroSet XT	Injectable, self-setting bone substitute composed of tetra-calcium phosphate that is formulated to convert to hydroxyapatite, the principal mineral component of bone.	Orthopedics Bone filler	[243]
DirectInject	The first and only on-demand, self-setting HAp cement. It is used to repair neurosurgical burr holes, contiguous craniotomy cuts and cranial defects.	Orthopedics Neurosurgery Bone filler	
Vitoss^®^	Beta-tricalcium phosphate and bioactive glass. Available in many forms such as moldable packs, malleable strips, and morsels.	Orthopedic Bone graft	
Osteoset^®^	Resorbable CaP ceramic that consists of synthetic calcium sulfate beads for bone grafting, calcium sulfate.	Bone graft	
Calcigen^®^	Synthetic calcium sulfate particles for bone grafting, calcium sulfate.	Bone Void Filler	[253]
ProOsteon^®^	Porous hydroxyapatite particles that are osteoconductive and have a structure and chemistry similar to human bone.	Orthopedics Bone grafts	
BonePlast^®^	Medical bone void fillers. Calcium sulfate powder, resorbable, extrudable, and moldable bone void fillers.	Bone graft Bone filler	
Norian ^®^SRS, Norian ^®^CRS	An injectable, moldable, and biocompatible bone void filler. It contains calcium phosphate powder and sodium phosphate.		[254]
ChronOSTM Inject	Synthetic calcium phosphate bone substitute, injectable, osteoconductive, and resorbable. Irregular bone defects can be completely filled. It consists of a brushite matrix and tricalcium phosphate granules.		
Neocement^®^	Calcium phosphate cement is intended for filling bone defects of the skeletal system.	Orthopedics Traumatology	[255]
Biopex^®^-R	Calcium phosphate cement which consists of powder and liquid components.	Bone tissue replacement.	[256]
Apaceram	Synthetic hydroxyapatite that has macro pores and micro pores. Macro pores are effective for new bone formation, while micro pores provide interconnectivity of the pores.		
Superpore	It has a unique “triple pore structure”. Contains APACERAM Type-AX to absorbable tricalcium phosphate ceramics.		
JectOS^®^ TCH TCP Dental HP	Partially biodegradable cement with a composition of 45% TCP and 55% DCPD. Used to fill cancellous bone defects.		[257]
CERAFORM^®^	Biocompatible synthetic biphasic ceramic made of hydroxyapatite and beta-tricalcium phosphate.		[258]
Cerasorb^®^	Resorbable, pure-phase β-tricalcium phosphate with an interconnecting, open multi-porosity.	Implantology, General grafting	[259]
Bonetree*^®^*	Octacalcium phosphate (OCP)-based synthetic *bone* substitute material.	Orthopedics Dentistry Bone graft	[260]
MBCP*^®^*	Bioactive mixture of highly crystalline HAp and β-TCP (Tri Calcium Phosphate)-.	Ortopedics Dentistry Bone graft	[261]

These commercial CaP composites are also reviewed and mentioned in other works [262,263,264].

## 6. Challenges

One of the primary challenges in utilizing bioactive ceramics for tissue engineering lies in optimizing their dissolution rates. Excessively slow degradation can disrupt the dynamic interaction between the material and surrounding bone, whereas overly rapid dissolution may hinder new bone formation. Stiller et al. [265] highlighted the necessity of fine-tuning dissolution behavior to promote bone regeneration without inducing adverse effects.

Equally critical is ensuring sufficient biocompatibility. Minimizing cytotoxic risks and maintaining appropriate biological activity are essential for patients’ safety post-implantation [266]. The development of safe implant materials, whether in the form of bone grafts, substitutes, surface coatings, or injectable hydrogels, requires the use of high-purity base materials and precisely controlled concentrations of doping elements.

Bone tissue engineering is inherently complex due to the variability in bone defects, differences in shape, size, location, and etiology across patients. Consequently, scaffolds, grafts, and fillers must be personalized to accommodate unique anatomical and functional demands, including complex geometries. This level of customization can be achieved through advancements in additive manufacturing (AM), which allow for the fabrication of highly porous, interconnected, and mechanically robust structures with uniform pore size distribution. AM techniques also facilitate the replication of bone’s natural structural and chemical features, thereby enhancing cellular integration and tissue regeneration.

In coatings, surface topography plays a key role—studies indicate that increased surface roughness improves osteoblast attachment. However, the optimal surface architecture and crystallinity of calcium phosphate-based (CaP) materials, which are detected by osteoclasts and influence their metabolic activity and resorption capacity, require further in vivo investigation. Moreover, CaP particles may enter cells, modulating cellular homeostasis and differentiation.

Similarly, the performance of bioglasses is highly composition-dependent. Elements such as lithium, silicon, boron, phosphorus pentoxide, and various dopants (e.g., Mg, Sr, and Zn) significantly influence bioactivity, degradation kinetics, and mechanical integrity. By tailoring these compositions, bioceramic materials can be customized for diverse medical applications. Further research should focus on elucidating the synergistic interactions among multiple dopants—especially in varying concentrations and ratios—within bioglass and CaP matrices, as these combinations hold potential to enhance tissue regeneration and wound healing outcomes.

Expanding clinical trials to assess the long-term performance of bioactive ceramic and bioglass-based implants is essential. Standardized, reproducible evaluation protocols are particularly critical for complex injuries involving both hard and soft tissues, where self-repair mechanisms are limited, and pathogenic factors complicate treatment.

Despite existing challenges, bioactive ceramics continue to demonstrate considerable potential for applications in the regeneration of bone, skin, tendons, cartilage, blood vessels, and myocardium. However, the development of bioceramic-based wound healing materials remains limited, with ongoing challenges that include the following:

- Variability in mechanical, chemical, and biological properties, driven by the type of bioceramic, biopolymer matrix, and fabrication methods.
- Difficulty in achieving precise control over scaffold vascularization, degradation kinetics, and standardized manufacturing processes.
- Limited reproducibility in the distribution of ceramic particles within polymer matrices.

To overcome these barriers, advanced and standardized protocols for material synthesis and processing are needed to improve implant performance and quality. In summary, key obstacles remain in achieving optimal mechanical strength, controlled biodegradability, and cost-effective scalability for widespread clinical use.

## 7. Future Perspectives

To date, the precise methods for regulating the types, dimensions, and forms of bioactive ceramics, along with their specific interactions with biological responses, remain poorly understood. Thus, future work must continue to refine these materials to optimize their mechanical properties, control degradation kinetics, and enhance biological performance for improved clinical outcomes. The enhancement of mechanical properties is especially critical for load-bearing applications, while optimized composition, interconnected and highly porous morphology are essential for delivering therapeutic ions or drugs incorporated into their network. Standardization of in vivo protocols and extended clinical trials will help clarify the long-term safety and efficacy of these versatile biomaterials.

Calcium phosphate-based ceramics (CaPs) are widely used in bone tissue engineering due to their bone-like composition, which confers excellent biocompatibility and osteoconductivity. With optimized chemical compositions, they have demonstrated tunable degradation rates beneficial for bone regeneration. In vitro studies consistently confirm their ability to support cell adhesion, proliferation, and osteogenic differentiation, while in vivo studies show gradual resorption and replacement by new bone tissue. However, further research is needed to optimize phase composition (e.g., tailoring Ca/P ratios and doping element concentrations), improve mechanical properties through composite formulations, and enhance biological performance via organic/inorganic additives or therapeutic agents.

In contrast, bioglasses, with their complex oxide-based compositions, are versatile for both soft and hard tissue engineering. Their chemical compositions, component ratios, and bioactive dopants can be flexibly adjusted to optimize biological characteristics and degradation rates. However, the molecular dynamics between cells and bioactive particles/bioglasses remain underexplored, yet this knowledge is crucial for maximizing their biological potential.

Regarding scaffold fabrication, increased adoption of advanced techniques like 3D/4D printing could enable patient-specific, highly porous scaffolds with controlled architectures. Future efforts should prioritize novel fabrication methods and explore synergistic combinations of bioactive ceramics and bioglasses to advance regenerative medicine.

Hybrid scaffolds combining CaPs, bioglasses, and polymers offer a straightforward and efficient strategy. Incorporating bioactive ions in carefully balanced amounts can enhance biological functionality, though further refinement is required. Notably, metallic ions (e.g., Mn^2+^, Cu^2+^, Fe^3+^, and Ag+) may trigger unexpected biological responses, necessitating deeper investigation. Understanding the long-term effects of these materials is vital for clinical translation, requiring more in vivo studies and clinical trials.

Given existing insights into CaP-based biomaterials, future innovations should focus on customizable designs, including tailored shapes and biodegradation rates [211]. Additionally, studies suggest that scaffold/implant surfaces mimicking native tissue microstructure, texture, and topography can enhance cell adhesion and organization. Future research should investigate cellular responses to nanoscale surface features and the underlying biological mechanisms.

## 8. Conclusions

This review discussed that bioglasses and calcium phosphate-based ceramics (CaPs) are not only highly biocompatible and osteoconductive but also possess tunable degradation profiles that can be matched to the rate of bone regeneration. Both in vitro and in vivo studies confirm their potential for diverse clinical applications in hard and soft tissue engineering, with primary uses including scaffolds, grafts, composites, hydrogels, and thin matrices. These materials have been shown to promote osteoconduction, osteoinduction, and antibacterial activity via controlled ion release, while clinical trials validate their efficacy in dentistry, craniofacial, orthopedic surgery (e.g., bone grafts, fillers, and injectable composites), wound healing, and drug delivery systems.

This review emphasized that in vivo and clinical studies consistently support bioactive ceramics as controllably degradable materials that stimulate bone regeneration and may exhibit antibacterial properties. Advances in fabrication technologies have significantly expanded the diversity of bioactive ceramics, enabling customization of composition, particle size, and morphology to suit specific tissue regeneration needs.

Key findings revealed that bioactive CaPs and bioglasses are promising for tissue engineering due to their unique bioactivity, biocompatibility, and mechanical adaptability. Their capacity to enhance bone regeneration, angiogenesis, and soft tissue repair positions them as versatile tools for clinical use. Notably, CaP-based materials are particularly suited to bone tissue engineering, as their calcium phosphate composition mimics the mineral phase of natural bone. In contrast, bioglasses, owing to their oxide-based structure and adaptable bioactivity, are effective in both soft and hard tissue repair, despite their inherent reactivity in biological systems.

In conclusion, the tunable degradation and biological responses of these materials make them ideal candidates for advancing regenerative medicine. Future efforts should focus on optimizing their design for targeted applications while addressing the remaining challenges in scalability and clinical translation.

## Figures and Tables

**Table 1:** Most used preparation methods for CaPs, classified as wet chemical methods and dry methods [21,22,23,24,25,26].

Wet methods	Wet chemical precipitation	Benefits	• Low cost • Low temperature • Simple procedure • Particle sizes and shapes can be controlled and altered via parameters • Environmentally friendly.
		Drawbacks	• Residual salt impurities • Imperfect solvent elimination • Difficult reproducibility • Mixed CaP phases can be obtained, and post-treatment is needed to obtain phase purity • Necessity of powder separation after precipitation.
	Sol–gel method	Benefits	• Low temperature • Simple procedure • High-quality final product • Homogeneous phase can be obtained • Controllable chemical composition.
		Drawbacks	• Expensive raw materials • Preparation parameters are difficult to control • Longer reaction time • Toxic organic solvents • Gel volume reduction (shrinking) after drying • Precursor agents • Sensitive to environmental conditions.
	Hydrothermal (aqueous)/Solvothermal (non-aqueous) synthesis	Benefits	• Simple and easy • Highly crystalline • Good control over size and morphology • High purity, Homogeneous phase products • Low agglomeration • Better nucleation control • Better shape control • Tunable chemical composition
		Drawbacks	• Relatively high temperatures • High energy consumption • High pressure • Equipment limitations • Reaction conditions needed to be precisely controlled • Longer reaction time • Limited scalability • Toxic organic solvents.
Dry methods	Mechano-chemical (mechanical forces induce chemical reactions and structural changes)	Benefits	• Simple and cost effective • Ambient condition • Cheap reactants • No harmful by-products • Utrafine and nanostructured CaP powders • Large surface area • Enhanced bioactivity • Uniform mixing of different precursor materials • Environmentally friendly.
		Drawbacks	• Easily contaminated • Limited phase control • Difficult to achieve phase-pure CaPs • Agglomeration and irregular morphology • Long processing time • High mechanical stress.
	Solid-state reaction: -decomposition -Chemical reaction between solids and reduction	Benefits	• High purity and crystallinity • Basic equipment • Cheaper than hydrothermal or sol–gel methods • Suitable for large-scale production • Different CaP phases by adjusting the temperature and reaction time, (hydroxyapatite, tricalcium phosphate, etc.).
		Drawbacks	• High energy consumption • Solid precursors might not mix uniformly • Phase inhomogeneities • Large particle size • Aggregation • Slow Reaction Kinetics • Long reaction times for complete phase transformation.

**Table 2:** Mechanical properties of the bioglass scaffolds, calcium phosphate-based scaffolds, and natural bones.

Properties	Bioglasses	Calcium Phosphates	Natural Bone (Cortical)
Compressive Strength	500–1000 MPa (dense) 2–100 MPa (porous)	500–600 MPa (dense HAp) 2–12 MPa (porous HAp/TCP)	100–230 MPa
Tensile Strength	40–60 MPa	10–100 MPa (dense HA) <5 MPa (porous)	50–150 MPa
Brittleness	High (ceramic, no collagen)	High (ceramic, porous scaffolds)	Low (collagen matrix absorbs energy)
Fracture Toughness	0.5–1 MPam^1/2^	~1 MPam^1/2^ (dense HAp)	2–12 MPam^1/2^
Hardness (Vickers)	3–5 GPa	3–5 GPa (HAp)	0.3–0.5 GPa
Durability	Degrades in weeks–months (bioactive)	TCP: months; HAp: years (depends on porosity)	Self-repairing; remodels lifelong

**Table 3:** Preparation methods of drug-loaded bioactive ceramics.

Method	Description
Impregnation	Loading drugs into pre-formed CaP scaffolds by soaking them in a drug-containing solution.
Co-precipitation	Simultaneous precipitation of CaP and the drug from a solution, leading to homogeneous distribution of the drug within the matrix.
Encapsulation	Enclosing drugs within CaP microspheres or nanoparticles providing controlled release profiles.

**Table 4:** Preparation methods of ceramic coatings onto implant materials and coating properties [139,140,141,142,143,144,145,146,147,148,149,150].

Technique	Coating Characteristics	
	Advantages	Disadvantages
Plasma Spray	Homogeneous and dense layer; fast deposition rate; coating thickness and deposition parameters are easy to control; good coating adherence to substrate; improved corrosion and wear resistance.	Expensive equipment; high temperature; crack development; complex-shaped substrates are difficult to coat.
Chemical Vapor Deposition		
Pulsed Laser Deposition		
Electrospraying/electrospinning	Nanofibrous; porous structure; high specific surface area; mimics ECM.	Poor adhesion; limited thickness; poor mechanical properties.
Spin/Dip coating	Smooth, thin film, good adhesion, moderate mechanical properties.	Low porosity; limited thickness.
Electrochemical deposition	Compact, crystalline coating, good adherence, tailored thickness, scalable coats on complex shapes.	Requires conductive substrate; limited polymer use; poor porosity and mechanical properties.
Electrophoretic deposition	Uniform and dense coating, good adhesion, moderate porosity, and scalable coats on complex shapes.	Cracking risk; requires stable suspension; poor mechanical properties.

**Table 5:** Different bioactive ion dopants and their effects on the base materials.

Element	Effect
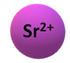	Influence both osteoblast (bone-forming cells) and osteoclast (bone-resorbing cells) activity.
	Repair bone defects and promote osseointegration.
	Mimic calcium ion (Ca^2+^) and modulate key signaling pathways involved in bone formation and resorption.
	Enhanced bone formation due to osteoblast stimulation.
	Reduced bone resorption by inhibiting osteoclastogenesis.
	Improved bone mineral density (BMD) and fracture healing.
	Favorable effects on metabolic energy balance, supporting bone tissue homeostasis.
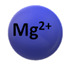	Enhance immunomodulation, angiogenesis, and vascularized osteogenesis in bone defect areas.
	Promote osteoblast proliferation and differentiation.
	Facilitate osteoblast adhesion and matrix mineralization.
	Accelerate HAp nucleation kinetics.
	Regulates calcium homeostasis, essential for hydroxyapatite formation.
	Inhibits osteoclast activity.
	Essential cofactor for ATP production, supporting osteoblast energy demands.
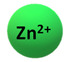	Enhance bone metabolism, cell proliferation, and tissue regeneration.
	Significant role in bone tissue’s normal development and maintaining homeostasis.
	Enhance ossification in stem cells.
	Promote osteogenesis and mineralization and confer antibacterial properties.
	Key transcription factor in osteoblast differentiation.
	Increasing osteogenic gene expression.
	Inhibits osteoclast differentiation.
	Boosts protein synthesis.
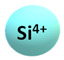	Facilitate human cell adhesion and differentiation.
	Induce angiogenesis, collagen type I, and osteocalcin expression.
	Enhance bone matrix quality.
	Promote osteoblast differentiation.
	Support hydroxyapatite crystallization and mineralization.
	Improve mitochondrial respiration in osteoblasts.
	Enhance nutrient transport via silicon-mediated ion exchange.
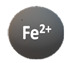	Essential for collagen cross-linking, aiding in bone matrix stability
	Promote vascularization in bone healing.
	Affecting osteoblast proliferation.
	Excess iron ions can increase oxidative stress, inducing osteoclastogenesis.
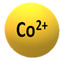	Promote angiogenesis.
	Improve vascularization in bone grafts.
	Stimulate osteogenic differentiation.
	Excess Co can cause oxidative stress, leading to cytotoxicity at high concentrations.
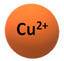	Stimulate angiogenesis.
	Increase collagen synthesis and cross-linking, improving bone matrix.
	crucial for bone stability.
	Excess Cu can generate reducing oxidative stress (ROS), potentially leading to cytotoxic effects.
	Slight antibacterial effect.
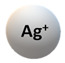	Exhibit antimicrobial properties, preventing infection in bone implants.
	Stimulates osteoblast proliferation at low concentrations.
	Inhibit osteoclast differentiation, balancing bone resorption.
	Disrupt bacterial metabolism without significantly affecting osteoblasts at low doses.
	Alter mitochondrial function, potentially inducing apoptosis at high concentrations.

**Table 6:** The biological effect of bioactive ion dopants on cell metabolisms.

Ions	Osteoblast Activity	Osteoclast Activity	Energy Metabolism
**Sr^2+^**	Activate Wnt/β-Catenin ^1^ signaling pathway → bone formation. Increase Osteoprotegerin (OPG ^2^) level Stimulate of MAPK/ERK ^3^ pathway Modulate of Calcium-Sensing Receptor (CaSR ^4^) Activate osteogenic genes such as Runx2 ^5^	Inhibit of RANKL ^6^ pathway → bone resorption Induce apoptosis in osteoclasts, further limiting bone resorption	Increase ATP ^7^ production Boost collagen and non-collagenous protein synthesis, essential for the formation of new bone
**Mg^2+^**	Activate Wnt/β-Catenin signaling pathway Enhance integrin binding, facilitating osteoblast adhesion and matrix mineralization Regulate calcium homeostasis	Inhibit of RANKL pathway	Increase ATP production Enhance glycolysis and oxidative phosphorylation Reduce oxidative stress by activating superoxide dismutase (SOD)
**Zn^2+^**	Stimulate osteogenic genes such as Runx2 Activate the MAPK/ERK pathway, increasing osteogenic gene expression Activate the MAPK/ERK pathway, increasing osteogenic gene expression	Inhibit osteoclast differentiation by reducing RANKL signaling	Boost protein synthesis by upregulating ribosomal function Increase antioxiant defenses via metallothioneins Enhance insulin-like growth factor (IGF-1) signaling, promoting osteoblast proliferation
**Si^4+^**	Induce collagen type I and osteocalcin expression, enhancing bone matrix quality Modulate TGF-β ^8^ and BMP ^9^ pathways, promoting osteoblast differentiation Support hydroxyapatite crystallization and mineralization	Minimal effect	Improve mitochondrial respiration in osteoblasts Enhance nutrient transport via silicon-mediated ion exchange Modulate reactive oxygen species (ROS) balance, protecting against oxidative damage
**Fe^2+^/Fe^3+^**	Influence Wnt and BMP pathways, affecting osteoblast proliferation Promote vascularization in bone healing Essential for collagen cross-linking, aiding in bone matrix stability	Cause osteoclastogenesis at high concentrations	Crucial for ATP production via electron transport chain Regulate oxygen metabolism, ensuring proper cell function
**Co^2+^**	Enhance VEGF ^10^ expression, improving vascularization in bone grafts Stimulate osteogenic differentiation via the BMP and MAPK pathways	Excess Co can cause oxidative stress, leading to cytotoxicity at high concentrations	Increase glycolytic metabolism in osteoblasts Affect iron homeostasis, altering mitochondrial function
**Cu^2+^**	Stimulate angiogenesis by upregulating VEGF Increase collagen synthesis and cross-linking, improving bone matrix Regulate lysyl oxidase activity which is crucial for bone stability	Excess Cu can generate ROS, potentially leading to cytotoxic effects	Enhance mitochondrial respiration and ATP production Act as a cofactor for superoxide dismutase (SOD), reducing oxidative stress
**Ag**	Exhibit antimicrobial properties, preventing infection in bone implants Stimulate osteoblast proliferation at low concentrations	Can inhibit osteoclast differentiation, balancing bone resorption, osteoclastogenesis	Disrupt bacterial metabolism without significantly affecting osteoblasts at low doses Alter mitochondrial function, potentially inducing apoptosis at high concentrations

^1^ The Wnt/β-catenin pathway comprises a family of proteins that play critical roles in embryonic development and adult tissue homeostasis. ^2^ OPG: Osteoprotegerin. ^3^ MAPK: Mitogen-Activated Protein Kinases. ^4^ CaSR: Calcium-Sensing Receptor. ^5^ Runx2: a gene that plays a cell proliferation regulatory role in cell cycle entry and exit in osteoblasts, as well as endothelial cells. ^6^ RANKL: (Receptor Activator of Nuclear Factor Kappa-B Ligand) an apoptosis regulator gene, a binding partner of osteoprotegerin (OPG), a ligand for the receptor RANK and controls cell proliferation. ^7^ ATP: Adenosine triphosphate. ^8^ TGF-β: Transforming growth factor beta, a cytokine, which regulates cell adhesion, proliferation, and differentiation. ^9^ BMP: Bone morphogenic protein. ^10^ VEGF: Vascular endothelial growth factor is a potent angiogenic factor. It is a signaling protein that promotes the growth of new blood vessels.

**Table 7:** Commercially available bioglass products and their main application fields.

Brand Name	Description	Application	Supplier
Medpor^®^-Plus™	Standard MEDPOR (biocompatible porous polyethylene particles) combined with bioactive glass (Bioglass^®^) mixture.	Orbital implants	[240]
NovaBone^®^ Perioglass	100% synthetic and resorbable calcium phosphosilicate dental putty.	Dentistry Orthopedics	[241]
Smart Healing™	S53P4 bioactive glass. Used for filling defects and replacing damaged bone tissue.	Orthopedics Bone filler Spine surgeries	[242]
Cortoss^®^	Injectable, bioactive composite material that mimics the mechanical properties of human cortical bone.	Orthopedics Osteoporosis	[243]
Glassbone™	Bioactive glass 45S5 ceramic composite used in regenerative medicine as a synthetic bioactive bone substitute.	Orthopedics Bone filler	[244]
StronBone™	Strontium containing bioactive ceramics or biomimetic fibrous polymer scaffolds.	Orthopedics Bone Graft Bone substitute Craniomaxillofacial	[245]
OssiMend^®^	Osteoconductive, bioactive bone graft matrix. Components: 50% carbonate apatite anorganic bone mineral, 30% 45S5 Bioactive Glass and 20% Type I Collagen	Orthopedics Spine surgery	[246]
Glace^TM^	Fiber-glass material that is used in post-traumatic surgery and for surgical bone reconstructions of the cranial and maxilofacial regions, including the orbital floor.	Orthopedics Spine surgery Orbital implants	[247]
Signafuse^®^	A composite of biphasic minerals and bioglass. Composition: bioglass and a biphasic mineral (60% hydroxyapatite, 40% β-tricalcium phosphate).	Orthopedics Spine surgery Cervical fusion Lumbar fusion Bone grafts	[248]
NovaMin^®^	The original *Bioglass^®^ 45S5*	Orthopedics Bone filler Bone graft	[249]
RediHeal™ RediHeal Ointment RediHeal Dental	Borate-based bioglass with unique trace elements. As an ointment, it treats topical soft tissue damage, minor abrasions, skin irritations, skin ulceration, burns, and scratches in humans and animals.	Veterinary Wound healing, ointment	[250]
OsteoGlass^®^	It has been designed with nano- and mesopores to promote osteoblast attachment and to allow blood vessels to grow through the scaffold and gradually degrade over the same timeframe as the new bone is formed.	Orthopedic Dental Skin treatment Wound healing	[251]

## Data Availability

The original contributions presented in this study are included in the article. Further inquiries can be directed to the corresponding author.
